# Syncope Associated With Alternating Fascicular Block in an Elderly Woman: A Case Report

**DOI:** 10.7759/cureus.105663

**Published:** 2026-03-22

**Authors:** Francesca Cali, Goitom Weldearegay, Chris Sani, Asher Gorantla, Adam S Budzikowski

**Affiliations:** 1 Internal Medicine, State University of New York Downstate Health Sciences University, Brooklyn, USA; 2 Internal Medicine, State University of New York Downstate Medical Center, Brooklyn, USA; 3 Cardiology, State University of New York Downstate Medical Center, Brooklyn, USA

**Keywords:** alternating fascicular block, bradyarrhythmia, bundle branch block, electrocardiogram (ecg/ekg), pacemaker (pm), permanent pacemaker implantation (ppi), presyncope, syncope

## Abstract

Alternating fascicular block (AFB) is defined by intermittent left anterior fascicular block and left posterior fascicular block on serial electrocardiograms. Although alternating bundle branch block is well recognized as a high‑risk conduction abnormality warranting permanent pacing, evidence and guideline clarity for AFB remain limited. This case describes an 81‑year‑old woman with recurrent syncope. AFB was found on EKG, and a permanent pacemaker was introduced. AFBs are a supporting marker of advanced infra‑Hisian conduction disease and can be associated with syncope and clinically significant bradyarrhythmias. In symptomatic patients, early consideration of permanent pacing is reasonable despite the absence of explicit guideline recommendations. This case highlights key diagnostic features and management considerations while underscoring the need for clearer evidence-based guidance regarding AFB.

## Introduction

Fascicular blocks result from impaired conduction within the anterior or posterior divisions of the left bundle branch. While an isolated fascicular block is often benign, an alternating fascicular block (AFB), manifested by intermittent left anterior fascicular block (LAFB) and left posterior fascicular block (LPFB) on serial electrocardiograms, could suggest diffuse and unstable disease within the His-Purkinje system. This pattern is rare and far less commonly described than alternating bundle branch block, which is a recognized Class I indication for permanent pacing in current guidelines, even without syncope [1]. AFB has rarely been described, with initial reports in clinical research only being from histological evaluations, which show significant fibrosis and calcification of the heart [2]. The clinical significance of AFB remains poorly defined, particularly in patients presenting with syncope. We present a case illustrating the diagnostic evaluation and management considerations in an elderly patient with symptomatic AFB.

## Case presentation

**Figure 1:**
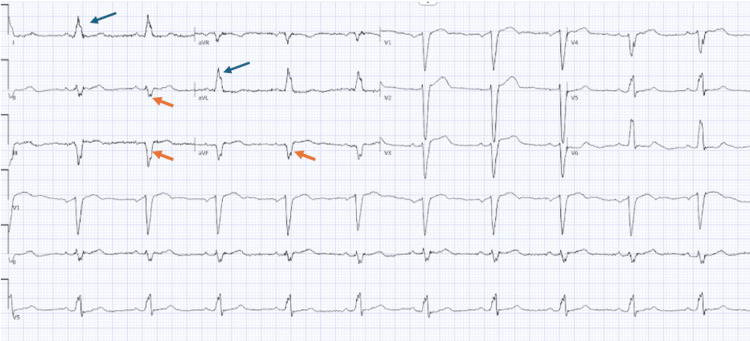
Electrocardiogram with left axis deviation, QRS duration of 126 milliseconds, and left anterior fascicular block Left anterior fascicular block caused by interrupted or delayed electrical conduction in the left anterior fascicle of the heart of the left bundle branch. Marked by qR (small q-wave and large R wave) (blue arrows) in leads 1 and aVL, as well as rS (small r-wave and large s-wave) (orange arrows) in leads 2, 3, and aVF.

An 81‑year‑old woman presents to the clinic with a history of hypertension presented for evaluation of recurrent presyncope and syncope. The patient reported a one‑year history of intermittent dizziness and near‑syncope, with occasional complete loss of consciousness. Symptoms most frequently occurred with positional change, particularly when standing, and had increased frequency over the preceding four weeks. Vital signs were within normal limits. Physical evaluation noted was grossly negative with normal heart sounds, clear airways, and no noted edema. Lab work, including a comprehensive metabolic panel, thyroid function tests, complete blood count, troponin, and pro-brain natriuretic peptide, was normal. Only magnesium was slightly elevated at 3.1 (range: 1.7-2.2 mg/dL). An EKG showed left axis deviation consistent with LAFB with slightly prolonged QRS (124 ms) (Figure 1).

**Figure 2:**
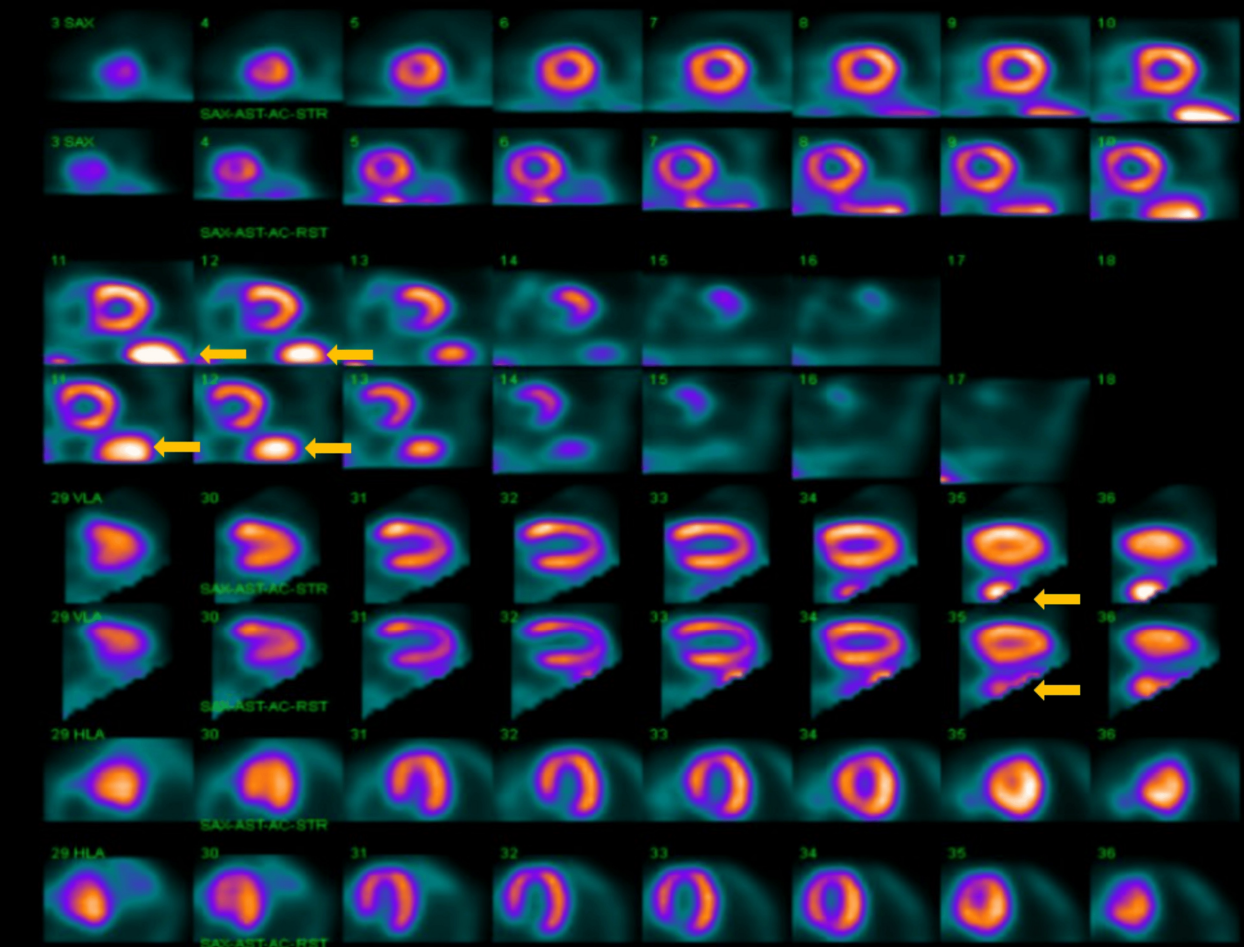
Single-photon emission computed tomography (SPECT) imaging with technetium showing no perfusion abnormalities, but visceral artifact is noted (yellow arrows) There are four sets of row, where the top row indicates perfusion in stressed heart, while lower row indicates perfusion in a resting heart. The first two rows are short axis images coming form the apex of the heart to the base, the third row is the vertical long axis view, and the fourth row is the horizontal long axis view.

The patient was not on any rate-controlling medications. She had prior testing for syncope, including negative tilt-table testing. A nuclear myocardial perfusion imaging (Figure 2) was done, which was grossly negative, except for some visceral artifact that was noted. Recent Holter monitoring showed bradycardia with pauses of up to 2.3 seconds, which correlated with pre-syncopal symptoms, as well as non-sustained ventricular tachycardia, which was noted to happen during sleep.

**Figure 3:**
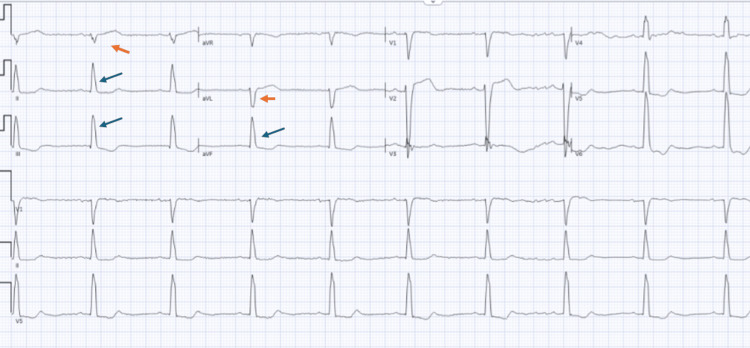
Electrocardiogram with sinus bradycardia, right axis deviation, QRS duration of 124, and left posterior fascicular block Left posterior fascicular block indicates delayed or blocked electrical conduction in the posterior division of the heart's left bundle branch. It is indicated by right axis deviation, rS (small r-wave and large s-wave) (blue arrows) complex in leads I and aVL and qR (small q-wave and large R wave) (orange arrows) complex in leads II, III, and aVF.

Review of electrocardiograms done during previous hospitalizations also noted an electrocardiogram with right axis deviation consistent with LPFB with slightly prolonged QRS (126 ms) (Figure 3). Transthoracic echocardiography showed normal left ventricular size and wall thickness, a visually estimated left ventricular ejection fraction of 65%, and abnormal septal bounce attributed to conduction abnormality.

In considering a combination of recurrent syncope episodes, symptomatic bradycardic and alternative fascicular blocks, and concern for advanced His-Purkinje disease, the patient was recommended for a pacemaker. Temporary measures, including monitoring and avoidance of AV-nodal blocking agents, were instituted while awaiting placement. Alternate options, including no pacemaker, leadless pacemaker, and continued observation with a loop recorder, were discussed. A dual‑chamber pacemaker was placed as decided by an electrophysiologist to preserve atrioventricular synchrony. After pacemaker implantation, the patient reported resolution of pre-syncopal episodes. Device interrogation at follow‑up demonstrated appropriate pacing during previously symptomatic pauses and no sustained ventricular arrhythmias. Long‑term follow‑up was arranged with the device clinic and cardiology.

## Discussion

This case underscores the clinical importance of AFB as a manifestation of His‑Purkinje system disease. While our patient, based on Holter monitoring, demonstrated symptomatic bradycardia and pauses, which could independently indicate a need for a pacemaker, there is no standardized evidence guiding management decisions specifically for AFB and indications for pacemaker placement. The 2018 American College of Cardiology/American Heart Association/Heart Rhythm Society (ACC/AHA/HRS) guidelines designate alternating bundle branch block as a Class 1 indication for permanent pacemaker implantation regardless of symptoms. Patients with bundle branch block and syncope are also candidates for pacemaker implantation if the His-ventricular (HV) interval exceeds 70 ms [1].

AFBs have rarely been described in the literature until recently, mostly in the context of a few case reports. However, one of the first papers that described it in a research setting was a 1979 histopathological study, which examined 13 hearts with LPFB, of which three patients demonstrated AFB primarily in the context of acute myocardial infarction. These patients who had chronic LPFB usually had extensive calcification on the left side of the heart [2]. Recent case reports have described an alternative fascicular block in the setting of severe pulmonary embolism from presumed pressure-induced His-Purkinje injury [3]. A similar recent case report described an AFB for which the patient underwent an electrophysiology study with a noted HV interval of 100, at which point a dual-chamber pacemaker was placed [4].

In a large-scale population study, fascicular block was found in 3.8% of patients. LPFB was the only block associated with increased risk of death, but isolated LPFB is rare [5]. As described in the previous study with AFB histopathology from 1979, LFPB and AFB were associated with extensive fibrosis of the left ventricle. Furthermore, both LAFB and LPFB are associated with increased risks of heart failure. Alternating bundle branch block reflects disease in the entire bundle branches, and this alternating effect is widely studied due to the significant risk of developing sudden-onset complete heart block [1]. The benefit of pacing supports consideration of consistent across different bundle branch block and fascicular block pathologies. In the Syncope: Pacing or Recording in the Later Years (SPRITELY) substudy, pacemaker placement in patients with bifascicular block, which is a combination of right bundle branch block with either LAFB or LPFB, showed a significant reduction in syncope recurrence, injuries, and further symptomatic bradycardia [6]. There are also prognostic indications for undergoing electrophysiology studies in patients with bifascicular block, as an HV interval greater than 60 milliseconds were independent predictor of subsequent need for pacemakers [7].

While most current data are centered around bi-fascicular blocks and alternating bundle branch blocks, we can use the data to extrapolate some of the effects of having AFB on patients. Considering that the histopathology of AFB showed worsening fibrosis and calcifications, our increasingly aging populations are thus at increased risk of the severity of conduction system disease [2]. Thus, more research is needed to understand the risks AFBs have on patients, which might involve the following questions: Are AFBs an indicator of severe infra-Hisian conduction disorders that will need early electrophysiological studies or early pacemaker implantation? Is there a high risk of progression to severe atrioventricular conduction disease?

## Conclusions

This case suggests that AFB may serve as a marker of diffuse and unstable intraventricular conduction disease and may not be a benign or incidental electrocardiogram finding. Although anatomically distinct from alternating bundle branch blocks, the two entities likely share important physiologic, clinical, and prognostic similarities, particularly regarding progression to advanced conduction diseases. In symptomatic patients - especially those with documented pauses - early pacing is a rational, protective strategy despite the lack of explicit guideline direction. Accordingly, AFBs should prompt heightened clinical vigilance and consideration of early electrophysiologic evaluation, and future guidelines should more explicitly address their role as a potential pacing indication.
